# Prevention of mammary carcinogenesis in rats by pregnancy: effect of full-term and interrupted pregnancy.

**DOI:** 10.1038/bjc.1988.88

**Published:** 1988-04

**Authors:** D. K. Sinha, J. E. Pazik, T. L. Dao

**Affiliations:** Department of Breast Surgery, Roswell Park Memorial Institute, Buffalo, New York 14263.

## Abstract

**Images:**


Br. J Cancer (1988), 57, 390-394 © The Macmillan Press Ltd., 1988

### Prevention of mammary carcinogenesis in rats by pregnancy: Effect of full-term and interrupted pregnancy[1]

D.K. Sinha, J.E. Pazik & T.L. Dao

*Department of Breast Surgery and Breast Cancer Research Unit, Roswell Park Memorial Institute, Buffalo, New York 14263 USA.*

**Summary** In this study, the role of parity in conferring protection of the mammary gland against chemical carcinogenesis induced by 7,12-dimethylbenz(a)anthracene (DMBA) was investigated. Experiments were also carried out to determine if an 'interrupted' pregnancy was capable of reducing the incidence of mammary tumour induction. Since it has been suggested that morphological development or the proliferative pattern of the mammary gland at the time of carcinogen administration may be involved in reducing the susceptibility of the mammary gland to chemical carcinogenesis, experiments were designed to elucidate the possible influence of these two factors. Sprague-Dawley female rats were mated and were either allowed to complete pregnancy and parturition or were subjected to Caesarian section on day 5, 10 or 15 of the pregnancy. When DMBA was administered i.v. to animals which had been allowed to complete a full-term pregnancy, only 14% developed tumours, compared to 70% in age-matched nulliparous controls. Termination of the pregnancy on days 5, 10 or 15 was as effective in reducing tumour incidence as full-term gestation and parturition, but still resulted in partial and statistically significant inhibition, compared to age-matched nulliparous controls. There was no significant difference in $^3$H-thymidine labelling index (LI) at the time of DMBA treatment in the parous rats compared to age-matched nulliparous controls. We also observed no significant differences in the morphological development of the mammary gland in parous and nulliparous rats of the same age. These results indicate that the protective mechanism may not lie in the mammary gland *per se*, but may indeed be a host factor, such as hormonal or immunological changes occurring in the host as a result of the pregnancy.

Epidemiologic observations have indicated that early full-term pregnancy renders breast tissue less susceptible to the action of a carcinogen (MacMahon *et al.*, 1973; Valaoras *et al.*, 1969) and late pregnancy and nulliparity have been shown to be associated with a higher risk of breast cancer (MacMahon *et al.*, 1973; Wynder *et al.*, 1960; Cutler, 1962). However, the mechanism by which parity confers refractoriness of the mammary gland to carcinogenesis is not clearly understood, although the epidemiologic studies demonstrating this effect have been mimicked, using experimental animals (Dao & Pazik, 1981; Russo & Russo, 1980). It has been shown that while nulliparous rats are susceptible to mammary carcinogenesis by 7,12-dimethylbenz[a]-anthracene (DMBA), parous rats of the same age are protected against the effect of the carcinogen. However, the relative importance of two factors, i.e., the age at which first pregnancy occurs and whether or not a full-term pregnancy is required to achieve the most effective degree of inhibition, remains unclear. Earlier reports from our laboratory have demonstrated that early full-term pregnancy in rats induced total protection of the mammary gland against carcinogenesis. The purpose of the present study was to further extend our investigation to elucidate the possible mechanism by which pregnancy inhibits mammary carcinogenesis, and to determine if a full-term pregnancy was required to induce refractoriness of the mammary gland.

### Materials and methods

Female and male Sprague-Dawley rats were obtained from Harlan Sprague-Dawley (Madison, WI) for this study. The animals were maintained in a temperature-controlled (24±1°C) room with a daily cycle of 14h light and 10h darkness. Females between the ages of 40–50 days were allowed to mate with 90-day-old male rats, three females being housed with one male. Mating started when the females were 40 days old and no rats over the age of 49 days were used for mating in these experiments. Vaginal smears were taken daily to determine when conception had occurred. The presence of a mucous plug or of sperm in the smear was taken as an indication of the exact day of conception. Pregnancy was subsequently confirmed by either parturition or uterine examination at the time of Caesarean section.

When pregnancy was established, the pregnant rats were placed in individual cages and divided into 5 groups. The rats in group no. 1 were allowed to complete gestation and parturition. The pups were removed at birth and the rats were allowed to rest for 15 days in order for involution of the mammary gland to occur. The animals in group no. 2 were treated similarly to those in group no. 1, except that they were allowed to lactate for five days following delivery. At the end of 5 days, the pups were removed and fifteen days were allowed for involution of the mammary gland to take place. In group no. 3, pregnancy was terminated by Caesarean section on the 5th day of pregnancy. In groups no. 4 and no. 5, pregnancy was terminated in the same manner on day 10 and day 15 of pregnancy, respectively. The animals in the three groups undergoing Caesarean section were also allowed 15 days following termination of the pregnancy, in order for involution of the mammary gland to occur. At the end of the 15-day resting period, all rats were given an i.v. injection of fat emulsion containing DMBA ($3\,mg^{-1}$ 100 g body wt). Controls were age-matched nulliparous females treated in the same manner as the parous rats. The animals were weighed and palpated weekly for tumours. At 120 days after treatment with DMBA, all animals were killed and autopsied, to determine if nonpalpable tumours were present. Tumours were excised and placed in Bouin's fixative for histological examination.

At the time of DMBA treatment, ten rats in each group, including the nulliparous controls, were sacrificed and the mammary glands fixed in alcohol-formalin-chloroform fixative (Sinha & Pazik, 1981) for wholemount preparation. The mammary labelling index was also determined at the time of DMBA administration, in order to indicate the level of DNA synthesis occurring in the gland at the time of carcinogen treatment. Three rats from each group, including the controls, were given an i.p. injection of 1 $\mu$Ci $^3$H-thymidine ($50\,Ci\,mmol^{-1}$, ICN Radiochemicals, Irvine, CA) per gram body wt. Two hours later, the animals were

Correspondence: D.K. Sinha.
Received 7 July 1987; and in revised form, 21 October 1987.

sacrificed. The right and left inguinal mammary glands from rats in oestrous were removed, fixed in Bouin's fluid and paraffin sectioned for autoradiography. NTB 2 (Eastman Kodak, Rochester, NY) was used to dip coat the slides. Labelling index was determined by counting the labelled cells compared to the total number of cells counted and was expressed as a percentage. A minimum of 3000 cells were counted from each gland. Statistical analysis was done either by Student's '*t*' test or '*V*' square, which is chi square test corrected for sample size (Rhoades & Overall, 1982).

### Results

### *Tumorigenesis*

There were three sets of age-matched nulliparous control rats. These rats had been placed in breeding cages with the males, but had never become pregnant. The control rats received DMBA at the ages of 69, 73 and 81 days, which corresponded to the ages at which the parous rats received the carcinogen. Of the 17 rats in group no. 5 (the 69-day controls), 15 (88%) developed tumours. In controls given the carcinogen at the age of 73 and 81 days, 18/21 (86%) and 26/27 (70%), respectively, developed tumours. Histologic examination demonstrated all the tumours to be adenocarcinomas.

Two groups of rats were allowed to complete a full-term pregnancy, but in one group, the rats were allowed to lactate for 5 days following delivery. The pups were then removed and the rats allowed to rest for 15 days prior to administration of DMBA. We observed no significant differences in tumorigenesis between the lactating or nonlactating groups. Of the rats which became pregnant between the ages of 40–46 days and were allowed to lactate for 5 days after parturition, 5/30 (16.6%) developed tumours. These rats were given DMBA at the age of 81 days. When similar rats without lactation were given DMBA, 5/37 (13.5%) developed tumours. Difference between these two groups were not statistically significant. Both groups were significantly different from the age-matched nulliparous controls, which demonstrated a 70% rate of tumour induction.

In group 3, pregnancy was terminated on the fifth day of pregnancy and the carcinogen was administered 15 days later. In this group, 13/27 (48%) developed tumours. When pregnancy was terminated on either day 10 or day 15, followed by carcinogen administration 15 days later, tumorigenesis was 12/24 (50%) and 14/31 (45%), respectively, whereas the age-matched nulliparous controls had a tumour incidence of 88% and 86%, respectively. This difference was also statistically significant (Table I, Figure 1). These data indicate that full-term pregnancy results in the greatest degree of tumour inhibition, since the incidence of mammary tumorigenesis was reduced from 70% in the controls to 14% in the full-term group. However, interrupted pregnancy also appeared to confer some degree of protection, since it resulted in a tumour incidence of 50% and 45%, as compared to 88% and 86% in nulliparous age-matched controls. There did not appear to be any discernible difference if the pregnancy was terminated at day 5, 10 or 15.

Average tumours per tumour bearing rats in nulliparous controls were 2.9 while in parous and parous lactating rats were 2.6 and 2.0 respectively. In the 5, 10 and 15 days C-section groups the average tumours/tumour bearing rats were 2.5, 2.0 and 2.6. The difference in number of tumour between groups was not statistically significant.

### *$^{3}$H-thymidine labelling index*

Table II summarizes the data on proliferative index of the mammary gland at the time of carcinogen treatment in the groups of animals described above. Since the rate of DNA synthesis has been shown to play an important role during carcinogenesis, we wished to determine if this factor was involved in the induction of refractoriness in parous rats. We found that the $^{3}$H-thymidine labelling index was high in the end buds of all groups used for these experiments, while that in the alveoli was consistently low. However, as indicated in Table II, there were no statistically significant differences in labelling index between any of the parous groups, as compared to the age-matched controls.

### *Morphology of the mammary gland*

Wholemount preparations were made at the time of carcinogen treatment in all groups. However, morphologic examination did not reveal any significant differences between the glands of parous and nulliparous animals of the same age. All wholemounts showed areas of ductal structures with club shaped end buds and areas where alveolar buds were predominant. Few end buds were observed in both types of glands, and the end buds appeared to be localized only in certain areas of the gland. The major part of the gland was composed of alveolar type of cells, which is typical of rats at that age (Figures 2–7). For numerical evaluation wholemount of 15 abdomino-inguinal mammary glands each from full-term parous rats and their age-matched nulliparous controls were evaluated. The end buds and the alveolar buds were counted per mm$^2$. This examination did not reveal any significant difference between the parous and nulliparous rats of same age. The mammary glands from parous rats showed 9.73 ($\pm$1.28) end buds and 36.13 ($\pm$2.10) alveolar buds. The mammary glands from nulliparous controls on the other hand showed 12.26 ($\pm$1.20) end buds and 32.00 ($\pm$2.10) alveolar buds per mm$^2$. The differences between the parous and nulliparous glands were not statistically significant.

**Table I** Tumorigenesis in mammary gland of rats after full-term and incomplete pregnancy

| *Term of pregnancy* | *No. of rats* | *Age at pregnancy (days)* | *Age at DMBA treatment (days)* | *No. of rats with tumours* | *Percent* |
|---|---|---|---|---|---|
| 5 days C-section | 27 | 49 | 69 | 13 | 48.1 |
| 10 days C-section | 24 | 45 | 70 | 12 | 50.0 |
| 15 days C-section | 31 | 40 | 70 | 14 | 45.1 |
| Full-term | 37 | 46 | 81 | 5 | 13.5 |
| Full-term/lactation | 30 | 46 | 86 | 5 | 16.7 |
| Age-matched control | 17 | – | 69 | 15 | 88.2 |
| Age-matched control | 21 | – | 73 | 18 | 86.7 |
| Age-matched control | 37 | – | 81 | 26 | 70.3 |

$P$=Full-term *vs*. Full-term lactation, 0.88; 5 days C-section *vs*. control, 0.007; 10 days C-section *vs*. control, 0.01; 15 days C-section *vs*. control, 0.03; Full-term *vs*. control, 0.004; Full-term lactation *vs*. control, 0.001.

**Figure 1** Tumour incidence in the mammary gland of parous rats after full-term (FT) or interrupted pregnancy. The numbers 5, 10 and 15 indicate the day on which pregnancy was terminated. Solid blocks represent age-matched nulliparous controls.

**Figure 2** Wholemount preparation of mammary gland from a parous rat at the time of carcinogen administration (81 days old). Note that the gland is mostly ductal, with some alveolar buds and very few terminal end buds present. H&E (×12).

**Figure 3** Wholemount preparation of mammary gland from an 81-day-old virgin rat. Note the similar morphological organization to that of the mammary gland of the parous animal, shown in **Figure 2**. The gland from the virigin animal is likewise mostly ductal with some alveolar buds and very few terminal end buds. H&E (×12).

**Figure 4** An enlarged area of the wholemount of mammary gland from parous rats showing predominantly ducts. Note the darkly stained (club shaped) end buds. H&E (×25).

**Figure 5** Enlarged area of the mammary wholemount from parous rat showing an alveolar area. H&E (×25).

**Figure 6** Enlarged view of ductal area of the mammary wholemount from age-matched virgin rats. H&E (×25).

**Figure 7** Enlarged area of an alveolar area from virgin control rat. H&E (×25).

**Table II** $^{3}$H-Thymidine labelling index of mammary glands from virgin, incomplete and full-term parous rats

| *Term of pregnancy* | *Age at labelling (days)* | *End buds* | *Alveoli* | *Average* |
|---|---|---|---|---|
| 5 days C-section[a] | 64 | 14.54±0.59[b] | 0.54±0.20 | 7.54±0.38 |
| Nulliparous controls | 64 | 15.75±2.84 | 1.61±0.64 | 8.57±1.80 |
| 10 days C-section[a] | 70 | 16.61±1.17 | 0.98±0.48 | 8.66±0.34 |
| Nulliparous controls | 70 | 11.30±0.93 | 1.64±0.38 | 6.47±0.63 |
| Full-term | 86 | 11.51±1.52 | 0.18±0.04 | 5.84±0.57 |
| Nulliparous | 86 | 10.32±1.88 | 0.58±0.04 | 5.27±0.62 |

[a]Labelling index was determined 15 days after termination of pregnancy or delivery.
[b]S.e.m.

### Discussion

The present experiments demonstrated that when rats completed a full-term pregnancy, mammary tumour induction was significantly reduced. This finding is in agreement with earlier studies by others (Cutler, 1962; Russo & Russo, 1980). Our studies also showed no significant difference in tumour incidence in the animals allowed to lactate following parturition as compared to tumour incidence in parous rats without lactation. Observations from similar experiments have been reported by others (Russo & Russo, 1980; Marchant, 1955; 1959). It should be noted that Russo and Russo (1980) observed the presence of more benign tumours in the groups of rats allowed to lactate. Marchant (1955, 1959) observed total inhibition of tumorigenesis in mice after lactation. None of these effects were demonstrated in our studies.

An interesting observation in our investigation is that termination of the pregnancy on day 5, 10 or 15 by Caesarian section failed to abolish the inhibitory effect of pregnancy on mammary tumour induction. In these rats, there was partial, but still significant, inhibition of subsequent DMBA-induced carcinogenesis. This result is in contradiction to the earlier work reported by Russo and Russo (1980), and the reasons for this discrepancy are not readily explained at this time. One possible explanation may be that a larger number of animals were used in our experiments, thus allowing us to observe an effect. Recent epidemiologic studies in humans have also reported conflicting results. Earlier investigations by Yuasa and MacMahon (1970) and Ravinhar *et al.* (1979) suggested that full-term pregnancy was required to achieve protection against breast cancer, but this was not supported by the later findings of Vessey *et al.* (1982). These authors have reported that incomplete pregnancy (termination by abortion) did not suppress the efficacy of pregnancy for protection of the mammary gland against carcinogenesis in humans. It would appear that our findings in experimental animals, in which we observed a partial reduction in tumour incidence in animals having incomplete pregnancy terminated by Caesarian section, are similar to those in man.

The mechanism by which pregnancy protects the mammary gland against carcinogenesis is not clearly understood. Russo and Russo (1980) suggested that morphological differentiation of the mammary gland confers protection against carcinogenesis. These authors hypothesized that during pregnancy terminal end buds and ductal buds become differentiated into alveolar buds which are known to be less susceptible to carcinogenesis. This suggestion, however, was not supported by our present findings, since we failed to observe any significant differences in morphological differentiation between the parous rats and their age-matched nulliparous counterparts. When wholemount preparations from these two groups of animals were compared, both showed the presence of mostly alveolar growth, and in both groups, there were very limited areas showing few terminal end buds. It would seem that predominately alveolar structure may be a characteristic of age, rather than a consequence of pregnancy. It should be noted that despite the similarities in morphology, the tumour incidence in the nulliparous rats was significantly higher than that in the age-matched parous animals.

The level of DNA synthesis at the time of carcinogen treatment has been shown to be an important factor influencing carcinogenesis (Lin *et al.*, 1976; Nagasawa & Vorherr, 1977). In the mitotically static mammary glands of older rats, tumour incidence was low, whereas in younger rats having higher levels of DNA synthesis in the mammary gland, the tumour incidence was significantly higher (Sinha *et al.*, 1983). Acceleration of DNA synthesis in the mammary gland of older rats could overcome the refractory effect of age, resulting in higher levels of tumour incidence (Sinha & Dao, 1980). However, in the present study, the level of DNA synthesis in the mammary gland of the parous rats at the time of carcinogen treatment did not show a statistically significant difference compared to that in nulliparous animals, although there was a significant difference in tumour incidence between the two groups. This was true for both the full-term and incomplete pregnancy groups, when compared to age-matched nulliparous controls. It appears that neither morphological changes nor levels of DNA synthesis in the mammary gland, as a result of pregnancy, exert an inhibitory effect on mammary carcinogenesis.

Vonderhaar and Topper (1974) have shown that pregnancy-type stimulation can produce a generation of cells in a mammary gland, which, after the completion of one mitotic cycle, continue to rest in the 'precritical' area of $G_1$ phase. If these cells are further stimulated, they are not obligated to go through a proliferative cycle; rather, they enter the secretory phase directly. The mammary cells of virgin female rats, on the other hand, rest in the 'postcritical' area of $G_1$ phase, and, upon further stimulation, are obligated to go through a mitotic cycle. This hypothesis would allow for meaningful interpretation of our data, since DMBA is not only a mammary carcinogen, but it can also stimulate mammary cells to replicate, as shown earlier by Sinha and Dao (1980). Since the cells in a parous mammary gland are resting in the 'precritical' area of $G_1$, treatment with DMBA would induce them to enter the secretory phase, but cell proliferation would not occur, and, thus, the neoplastic changes induced by the carcinogen would not be expressed.

Another possible explanation that might account for the lowered susceptibility of parous rats to chemical carcinogenesis is the fact that immunological changes are induced in the mother as a result of pregnancy. Conception results from allogenic matings and the embryo is in effect an 'allograft' in the mother. Thus, foreign antigens have been detected in embryos (Simmons & Russell, 1966; Heyner, 1973; Patthey & Edidin, 1973); in placental cells (Sellens *et al.*, 1978; Wegmann *et al.*, 1979); and in trophoblasts (Loke *et al.*, 1971). One category of antigens, designated as 'oncofetal antigens', such as alpha foetoprotein, chorionic gonadotropin, and chorionic somatomammotropin, also result in stimulus to the mother. In addition, recent investigations have revealed that several other antigens are produced by both the foetus and/or placenta and also by breast tumours. These would include pregnancy associated alpha-2 glycoprotein (Sarcione *et al.*, 1983); pregnancy-specific beta-1 glycoprotein (Grudzinskas *et al.*, 1980); placental lactogen (Monteiro *et al.*, 1982); and placental protein 5 (Bremner *et al.*, 1981).

Thus, it appears reasonable to suggest that both foeto-placental tissue and mammary tumours may produce some antigens in common, against which the mother has been immunized as a result of the pregnancy. This immunity may well be maintained in the mother after the pregnancy has been completed. It is our hypothesis that antibodies against some specific substances produced by breast tumours, being present in the parous female rat, are capable of recognizing the newly transformed mammary cells and act against those cells, thus providing an immunosurveillance mechanism which would 'protect' the parous female rat against subsequent tumour development. Our laboratory is now investigating the possible mechanisms by which this might occur.

This research was supported by Public Health Service Grant No. CA 36139 from the National Cancer Institute.

The authors are indebted to Patricia N. Coughlin for her assistance in the preparation of the manuscript.

### References

BREMNER, R.D., NISBET, A.D., HERRIOT, R. & 4 others (1981). Detection of placental protein five (PP5) and pregnancy-specific glycoprotein (SP 1) in benign and malignant breast disease. *Oncodevelop. Biol. Med.*, **2**, 55.

CUTLER, M. (ed) (1962). *Etiology, Tumors of the Breast*. J.B. Lippincott: Philadelphia.

DAO, T.L. & PAZIK, J. (1981). Early pregnancy protects against mammary gland carcinogenesis. *Proc. Am. Assoc. Cancer Res.*, **22**, 96 (*Abstract*).

GRUDZINSKAS, J., COOMBES, R., RATCLIFFE, J.G. & 4 others (1980). Circulating levels of pregnancy specific $\alpha_1$ glycoprotein in patients with testicular, bronchogenic and breast carcinomas. *Cancer*, **45**, 102.

HEYNER, S. (1973). Detection of H-2 antigens on the cells of the early mouse embryo. *Transplantation*, **16**, 675.

LIN, F.L., BANERJEE, M.R. & CRUMP, L.R. (1976). Cell cycle-related hormone carcinogen interaction during chemical carcinogen induction of nodule-like mammary lesion in organ culture. *Cancer Res.*, **36**, 1607.

LOKE, Y., JOYSEY, V. & BORLAND, R. (1971). HL-A antigens on human trophoblast cells. *Nature*, **232**, 403.

MACMAHON, B., COLE, P. & BROWN, J. (1973). Etiology of human breast cancer: A review. *J. Natl Cancer Inst.*, **50**, 21.

MARCHANT, J. (1955). Influence of pregnancy and lactation on the incidence of mammary carcinoma induced with methylcholanthrene in female mice of the IF strain. *J. Pathol. Bacteriol.*, **70**, 415.

MARCHANT, J. (1959). Local inhibition by lactation of chemically induced breast tumours in mice of IF strain. *Nature*, **183**, 629.

MONTEIRO, J., BISWASS AL-AWQUATI, M.A., GREENING, W.P., MCKINN, J.A. & NEVILLE, A.M. (1982). Serum levels of human placental lactogen and pregnancy-specific $\alpha_1$-glycoprotein in breast cancer. *Br. J. Cancer*, **46**, 279.

NAGASAWA, H. & VORHERR, H. (1977). Rat mammary deoxyribonucleic acid synthesis during the estrous cycle, pregnancy and lactation in relation to mammary tumorigenesis. Its implications for human breast cancer. *Amer. J. Obstet. Gynecol.*, **127**, 590.

PATTHEY, H.C. & EDIDIN, M. (1973). Evidence for the time of appearance of H2 antigens in mouse development. *Transplantation*, **15**, 211.

RAVINHAR, B., MACMAHON, B. & LINDTNER, J. (1979). Epidemiologic features of breast cancer in Slovenia, 1965–67. *Eur. J. Cancer*, **7**, 295.

RHOADES, H.M. & OVERALL, J.E. (1982). Quantitative methods. *Psychol. Bull.*, **91**, 418.

RUSSO, J. & RUSSO, I.H. (1980). Susceptibility of the mammary gland to carcinogenesis. II. Pregnancy interruption as a risk factor in tumor incidence. *Am. J. Pathol.*, **100**, 497.

SARCIONE, E.J., DELLUMO, D. & ZLOTY, M. (1983). Pregnancy-associated alpha-2 glycoprotein (2 PAG) synthesis by human breast cancer tissue and cultured cell lines. *Int. J. Cancer*, **31**, a143.

SELLENS, M.H., JENKINSON, E.J. & BILLINGTON, W.D. (1978). Major histocompatibility complex and non-major histocompatibility complex antigens on mouse ectoplacental cone and placental trophoblastic cells. *Transplantation*, **25**, 173.

SIMMONS, R.L. & RUSSELL, P.S. (1966). The histocompatibility antigens of fertilized mouse eggs and trophoblast. *Ann. N.Y. Acad. Sci.*, **129**, 35.

SINHA, D. & DAO, T.L. (1980). Induction of mammary tumours in aging rats by 7,12-dimethylbenz(a)anthracene: Role of DNA synthesis during carcinogenesis. *J. Natl Cancer Inst.*, **64**, 519.

SINHA, D. & PAZIK, J. (1981). Tumorigenesis of the mammary gland by 7,12-dimethylbenz(a)anthracene during pregnancy: Relationship with DNA synthesis. *Int. J. Cancer*, **27**, 807.

SINHA, D.K., PAZIK, J.E. & DAO, T.L. (1983). Progression of rat mammary development with age and its relationship to carcinogenesis by a chemical carcinogen. *Int. J. Cancer*, **31**, 321.

VALAORAS, V.G., MACMAHON, B., TRICHOPOULOS, D. & PILYCHRONOPOULOU, A. (1969). Lactation and reproductive histories of breast cancer patients in greater athens, 1965–1967. *Int. J. Cancer*, **4**, 350.

VESSEY, M.P., MCPHERSON, D., YEATES, D. & DOLL, R. (1982). Oral contraceptive use and abortion before first term pregnancy in relation to breast cancer risk. *Br. J. Cancer*, **45**, 327.

VONDERHAAR, B.K. & TOPPER, Y.J. (1974). Role of the cell cycle in hormone-dependent differentiation. *J. Cell Biol.*, **63**, 707.

WEGMANN, T.G., MOSMANN, T.R., CARSON, G.A., OLIJNYK, O. & SINGH, B. (1979). The ability of the murine placenta to absorb monoclonal antifetal H-2K antibody from the aternal circulation. *J. Immunol.*, **123**, 1020.

WYNDER, E.L., BROSS, I.J. & HIRAYAMA, T. (1960). A study of the epidemiology of cancer of the breast. *Cancer*, **13**, 559.

YUASA, S. & MACMAHON, B. (1970). Lactation and reproductive histories of breast cancer patients in Tokyo, Japan. *Bull. WHO*, **42**, 195.


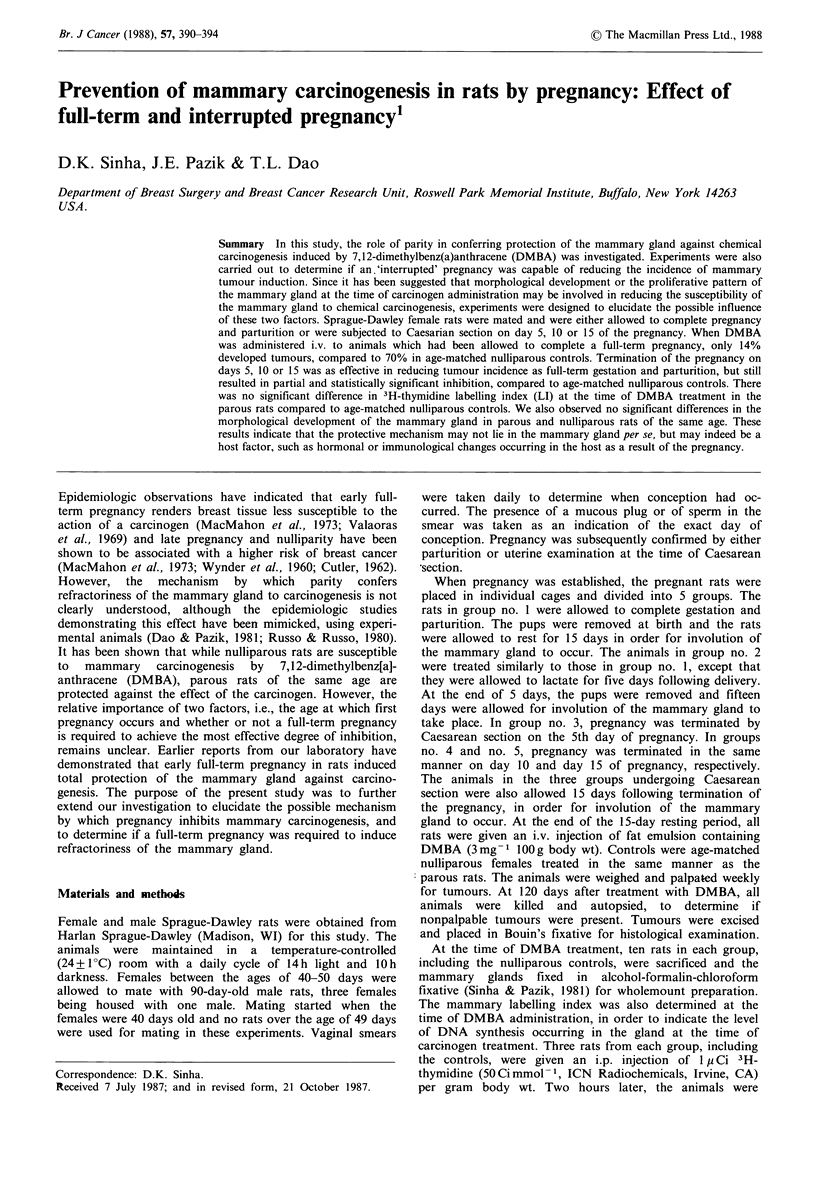

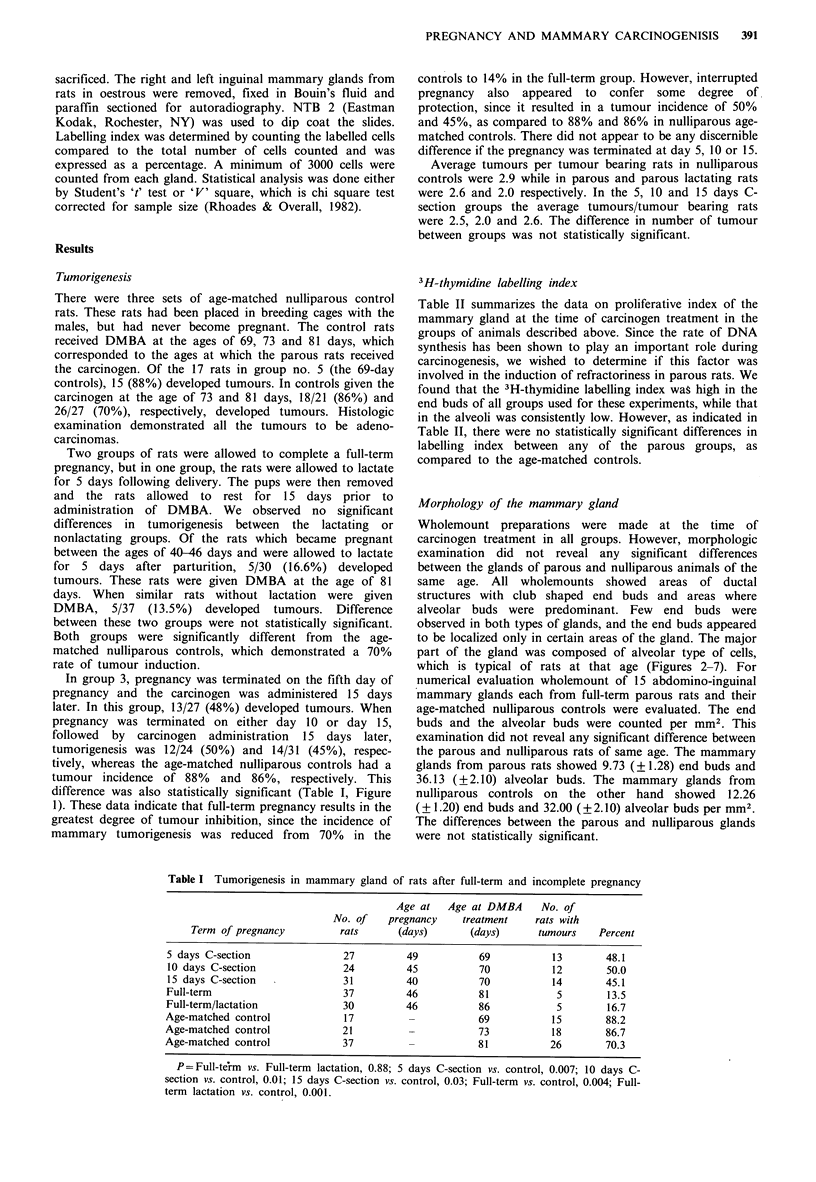

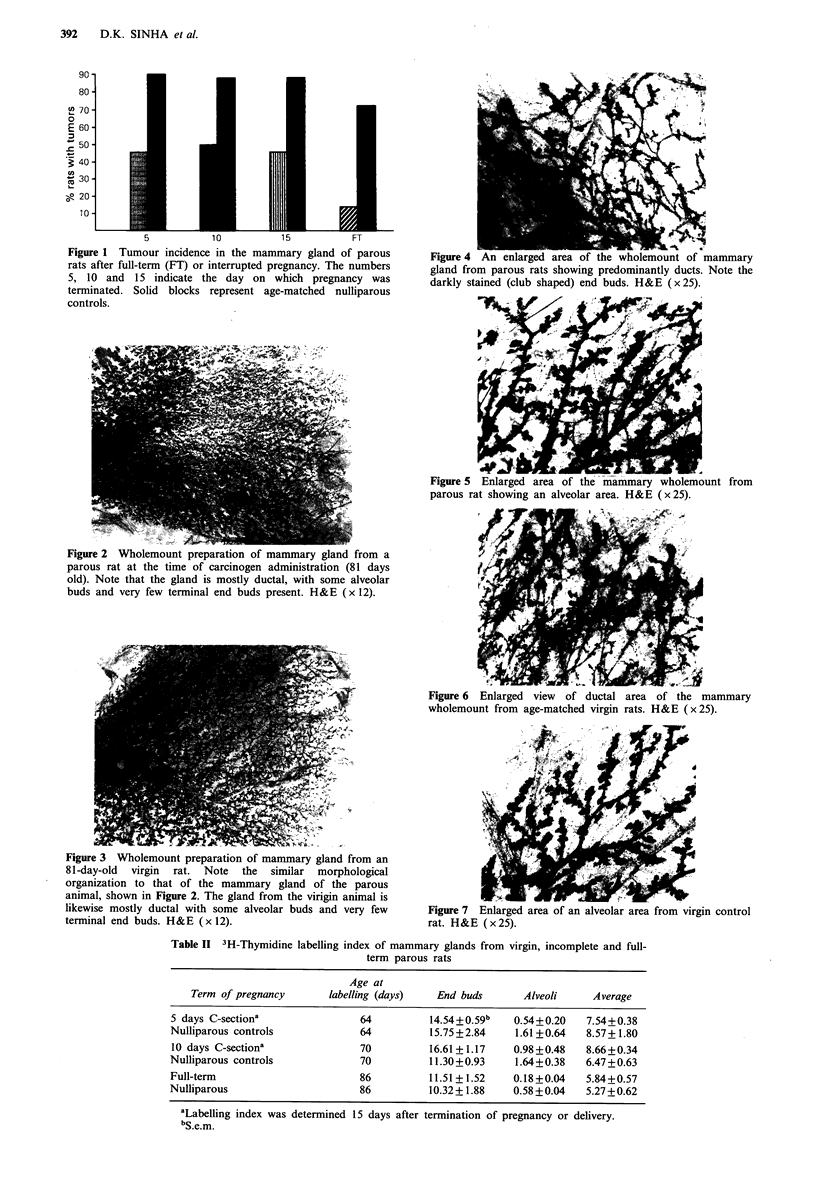

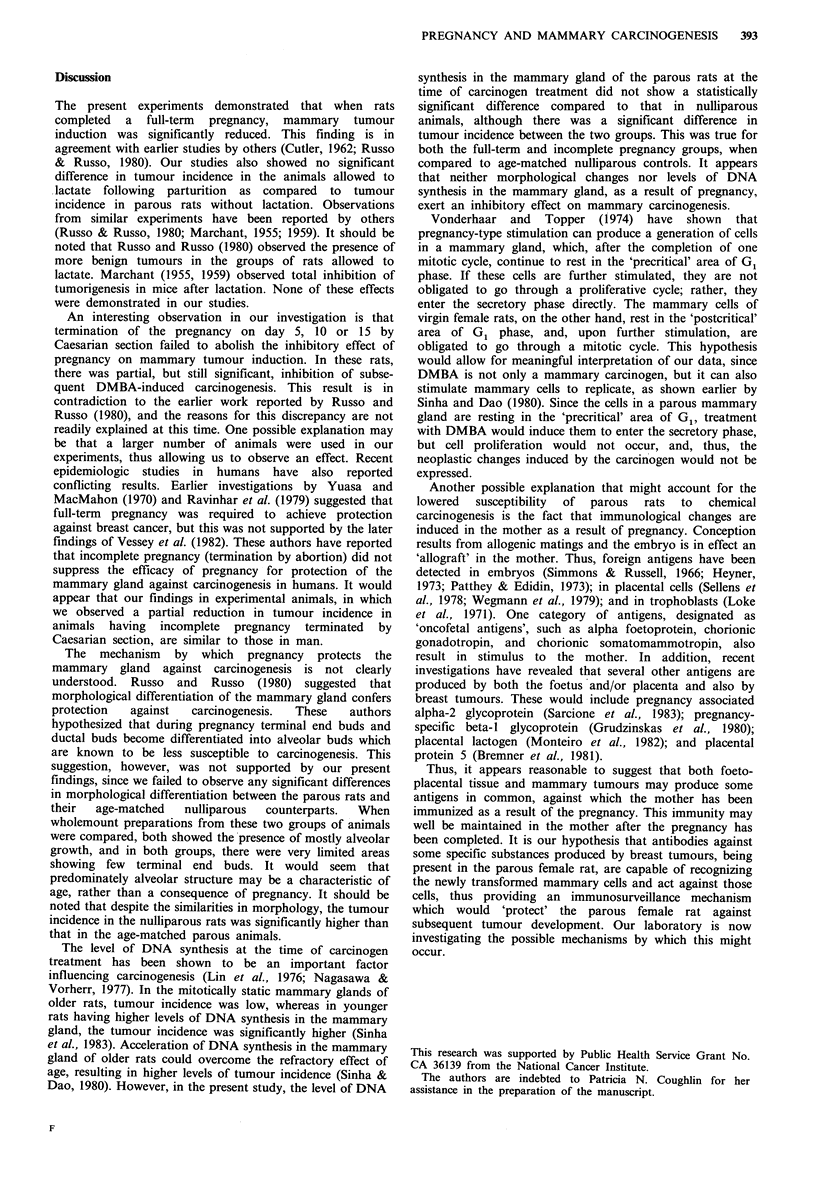

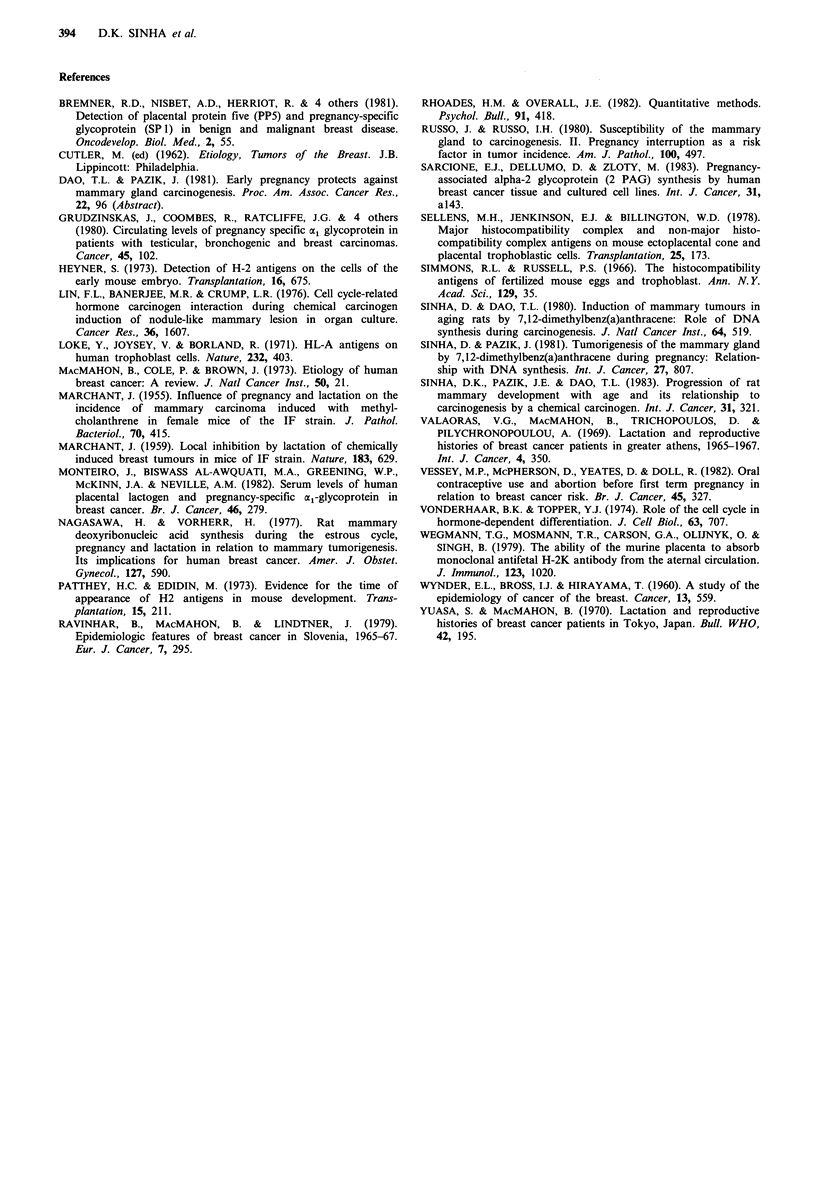


## References

[OCR_00513] Grudzinskas J. G., Coombes R. C., Ratcliffe J. G., Gordon Y. B., Powles T. J., Neville A. M., Chard T. (1980). Circulating levels of pregnancy specific beta 1 glycopretein in patients with testicular, bronchogenic and breast carcinomas.. Cancer.

[OCR_00519] Heyner S. (1973). Detection of H-2 antigens on the cells of the early mouse embryo.. Transplantation.

[OCR_00523] Lin F. K., Banerjee M. R., Crump L. R. (1976). Cell cycle-related hormone carcinogen interaction during chemical carcinogen induction of nodule-like mammary lesions in organ culture.. Cancer Res.

[OCR_00529] Loke Y. W., Joysey V. C., Borland R. (1971). HL-A antigens on human trophoblast cells.. Nature.

[OCR_00537] MARCHANT J. (1955). Influence of pregnancy and lactation on the incidence of mammary carcinoma induced with methylcholanthrene in female mice of the "IF" strain.. J Pathol Bacteriol.

[OCR_00543] MARCHANT J. (1959). Local inhibition by lactation of chemically induced breast tumours in mice of the IF strain.. Nature.

[OCR_00533] MacMahon B., Cole P., Brown J. (1973). Etiology of human breast cancer: a review.. J Natl Cancer Inst.

[OCR_00548] Monteiro J. C., Biswas S., Al-Awqati M. A., Greening W. P., McKinna J. A., Neville A. M. (1982). Serum levels of human placental lactogen and pregnancy-specific beta 1-glycoprotein in breast cancer.. Br J Cancer.

[OCR_00552] Nagasawa H., Vorherr H. (1977). Rat mammary deoxyribonucleic acid synthesis during the estrous cycle, pregnancy, and lactation in relation to mammary tumorigenesis: its implication for human breast cancer.. Am J Obstet Gynecol.

[OCR_00559] Patthey H., Edidin M. (1973). Evidence for the time of appearance of H-2 antigens in mouse development.. Transplantation.

[OCR_00564] Ravnihar B., MacMahon B., Lindtner J. (1971). Epidemiologic features of breast cancer in Slovenia, 1965-1967.. Eur J Cancer.

[OCR_00573] Russo J., Russo I. H. (1980). Susceptibility of the mammary gland to carcinogenesis. II. Pregnancy interruption as a risk factor in tumor incidence.. Am J Pathol.

[OCR_00584] Sellens M. H., Jenkinson E. J., Billington W. D. (1978). Major histocompatibility complex and non-major histocompatibility complex antigens on mouse ectoplacental cone and placental trophoblastic cells.. Transplantation.

[OCR_00595] Sinha D. K., Dao T. L. (1980). Induction of mammary tumors in aging rats by 7,12-dimethylbenz[a]anthracene: role of DNA synthesis during carcinogenesis.. J Natl Cancer Inst.

[OCR_00605] Sinha D. K., Pazik J. E., Dao T. L. (1983). Progression of rat mammary development with age and its relationship to carcinogenesis by a chemical carcinogen.. Int J Cancer.

[OCR_00600] Sinha D. K., Pazik J. E. (1981). Tumorigenesis of mammary gland by 7,12-dimethylbenz(a)anthracene during pregnancy: relationship with DNA synthesis.. Int J Cancer.

[OCR_00609] Valaoras V. G., MacMahon B., Trichopoulos D., Polychronopoulou A. (1969). Lactation and reproductive histories of breast cancer patients in greater Athens, 1965-67.. Int J Cancer.

[OCR_00615] Vessey M. P., McPherson K., Yeates D., Doll R. (1982). Oral contraceptive use and abortion before first term pregnancy in relation to breast cancer risk.. Br J Cancer.

[OCR_00620] Vonderhaar B. K., Topper Y. J. (1974). A role of the cell cycle in hormone-dependent differentiation.. J Cell Biol.

[OCR_00630] WYNDER E. L., BROSS I. J., HIRAYAMA T. (1960). A study of the epidemiology of cancer of the breast.. Cancer.

[OCR_00624] Wegmann T. G., Mosmann T. R., Carlson G. A., Olijnyk O., Singh B. (1979). The ability of the murine placenta to absorb monoclonal anti-fetal H-2K antibody from the maternal circulation.. J Immunol.

[OCR_00634] Yuasa S., MacMahon B. (1970). Lactation and reproductive histories of breast cancer patients in Tokyo, Japan.. Bull World Health Organ.

